# Biloma: A Rare Manifestation of Spontaneous Bile Leak

**DOI:** 10.7759/cureus.8116

**Published:** 2020-05-14

**Authors:** Muhammad N Yousaf, Rowena G D'Souza, Fizah Chaudhary, Hamid Ehsan, Charmian Sittambalam

**Affiliations:** 1 Internal Medicine, MedStar Union Memorial Hospital, Baltimore, USA; 2 Internal Medicine, MedStar Franklin Square Medical Center, Baltimore, USA; 3 Internal Medicine, MedStar Good Samaritan Hospital, Baltimore, USA; 4 Section of Digestive Diseases, Yale University School of Medicine, New Haven, USA; 5 Medicine, MedStar Union Memorial Hospital, Baltimore, USA; 6 Geriatrics, MedStar Franklin Square Medical Center, Baltimore, USA

**Keywords:** : biloma, spontaneous bile leak, pancreatitis, hepatobiliary interventions, percutaneous drainage, ercp

## Abstract

A biloma is an intrahepatic or extrahepatic encapsulated collection of bile outside of the biliary tree and within the abdominal cavity. Hepatobiliary interventions and laparoscopic cholecystectomy are the most common etiologies of biloma followed by abdominal trauma, choledocholithiasis, and biliary dilation secondary to biliary stricture. We report a case of a 91-year-old female who presented to the emergency room with an acute onset of epigastric and right upper quadrant sharp pain for one day that radiated to the back, and was associated with two to three episodes of vomiting. Initial abdominal imaging including CT scan, ultrasound and magnetic resonance cholangiopancreatography (MRCP) of the abdomen and pelvis revealed a distended gallbladder with wall thickening, but without evidence of pancreatitis or gallstones. Hepatobiliary iminodiacetic acid (HIDA) scan findings were consistent with extrahepatic biliary leakage into the peritoneum. A cholangiogram demonstrated a perihepatic biloma. A combined approach using fluoroscopic-guided biloma drainage and endoscopic retrograde cholangiopancreatography (ERCP)-guided biliary stent placement across the site of the biliary leak resulted in the complete resolution of symptoms. Biloma should be included in the differential diagnosis of right upper quadrant abdominal pain. A high index of clinical suspicion is required for early diagnosis and treatment.

## Introduction

A biloma is an encapsulated collection of bile outside the biliary tree and within the abdominal cavity. It can either be intrahepatic or extrahepatic. It is a rare condition with an incidence of 0.3% to 2.0% [1]. The most common etiologies include choledocholithiasis, abdominal trauma, and iatrogenic causes, such as postlaparoscopic cholecystectomy or hepatobiliary interventions [2]. A rare etiology of biloma is a spontaneous bile leak (SBL), where a specific cause remains unidentifiable and is usually a diagnosis of exclusion [3]. The clinical presentation of a biloma is variable, ranging from an incidental finding on imaging in an otherwise asymptomatic patient, to abdominal fullness, pain, fever, and jaundice, to very rarely, peritonitis without fever [3]. Abdominal imaging, such as abdominal ultrasound (US), CT scan, magnetic resonance cholangiopancreatography (MRCP), and cholescintigraphy using 99mTc hepatobiliary iminodiacetic acid (HIDA) scan, plays a crucial role in the identification of a biloma and in ruling out other possible etiologies [4]. The sensitivity of abdominal US is low (70%), though it is initial imaging in the evaluation of biloma [5]. The sensitivity and specificity of CT scan are approximately 90% and those of MRI are above 95% in the detection of biloma and bile leak; however, smaller bilomas can be missed [6]. HIDA scan is 97% sensitive, 94%-100% specific, and is the diagnostic modality in the detection of SBL [7]. Although HIDA scan is superior to both US and CT scan in the detection of bile leak, its utilization in conjunction with US and CT scan better delineates the anatomy of biliary tree [5]. With the advancement of endoscopic interventions, there is increasing use of endoscopic ultrasound (EUS) in the diagnosis and the management of spontaneous biloma. The rarity with which this occurs makes it an uncommon etiology on the differential of the epigastric and right upper quadrant (RUQ) abdominal pain. 

## Case presentation

**Figure 1:**
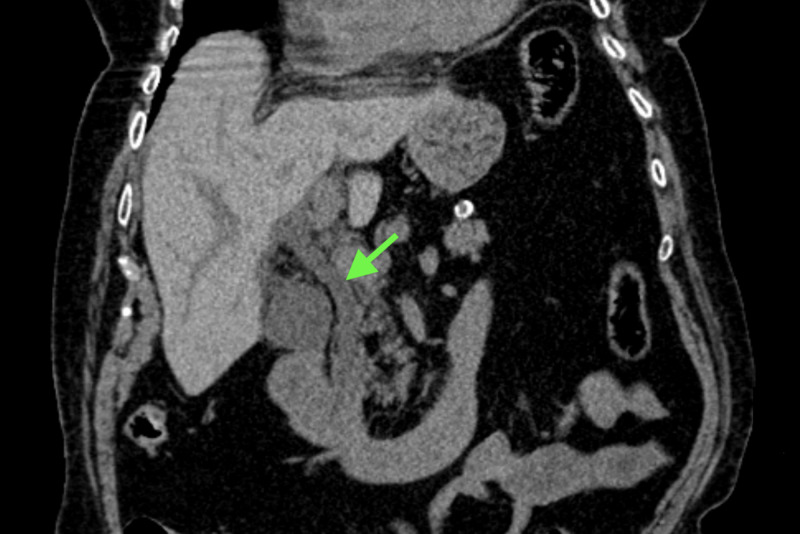
CT of the abdomen CT scan of the abdomen (coronal section) showing the dilated common bile duct (arrow) without clear peribiliary duct bile collection.

A 91-year-old female presented to the emergency room with acute onset of epigastric and RUQ pain for one day. The pain was described as sharp, radiating to her back, and associated with two to three episodes of non-biliary, non-bloody vomiting. She denied fever, bowel movement irregularities, weight loss, abdominal trauma, or prior abdominal surgeries. Clinical examination showed RUQ tenderness without rebound tenderness or Murphy’s sign. Laboratory workup was significant for elevated levels of lipase (5,700 U/L), lactic acid (2.2 mmol/L), and creatinine (1.71 mg/dL). A complete blood count, liver function tests (total bilirubin 0.2 mg/dL, alanine aminotransferase 9 U/L, aspartate aminotransferase 12 U/L, alkaline phosphate level 94 U/L), and alpha-fetoprotein (AFP) tumor (1.3 IU/mL) were unremarkable. CT scan of the abdomen and pelvis revealed a distended gallbladder with wall thickening, dilated common bile duct (CBD), but without evidence of pancreatitis or gallstones (Figure 1).

**Figure 2:**
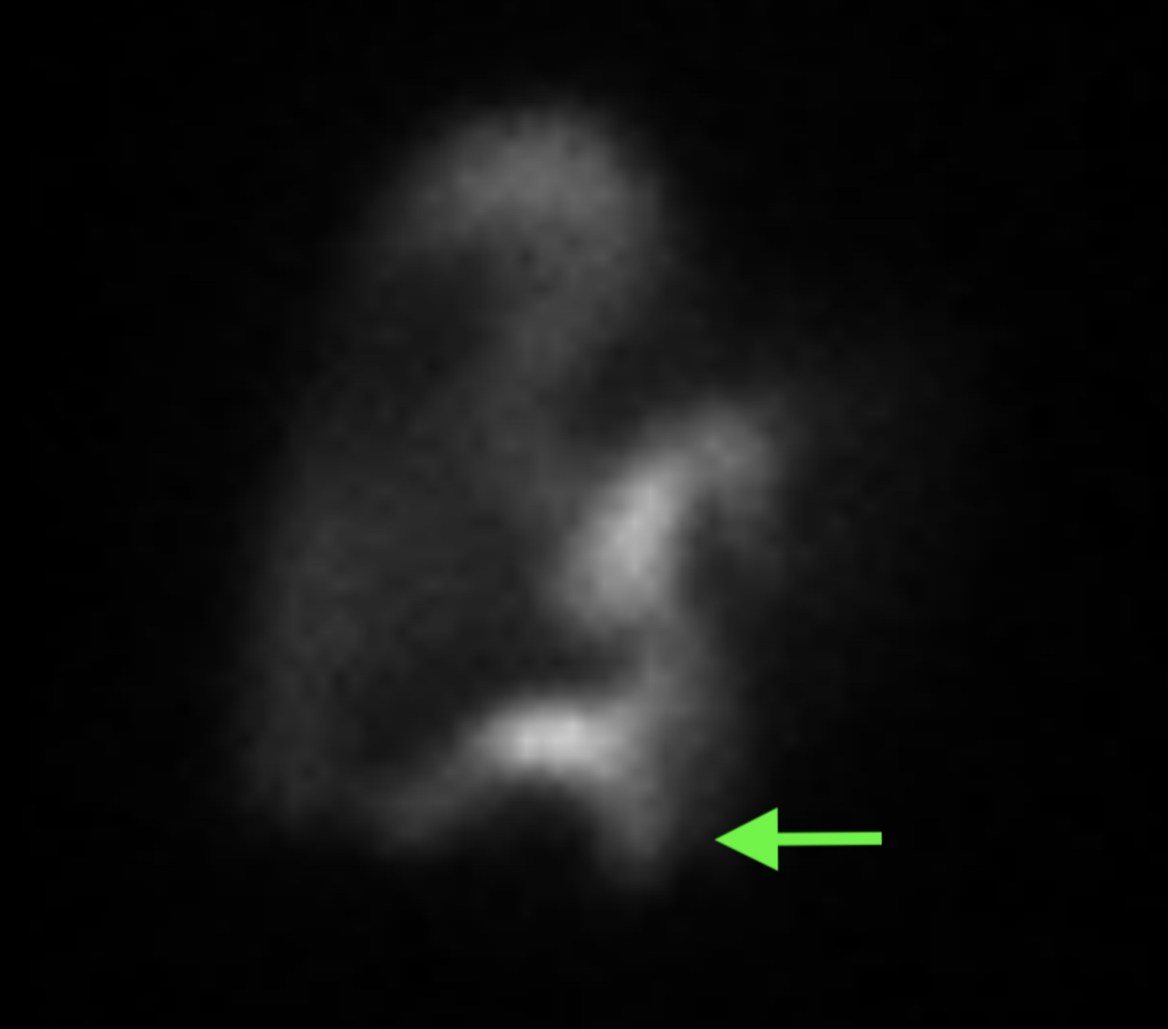
99mTc hepatobiliary iminodiacetic acid (HIDA) scan HIDA scan demonstrating tracer leak (arrow) indicative of bile leak.

Abdominal US showed trace intramural and pericholecystic fluid with no abnormality of the CBD. HIDA scan findings were consistent with extrahepatic biliary leakage into the peritoneum (Figure 2).

**Figure 3:**
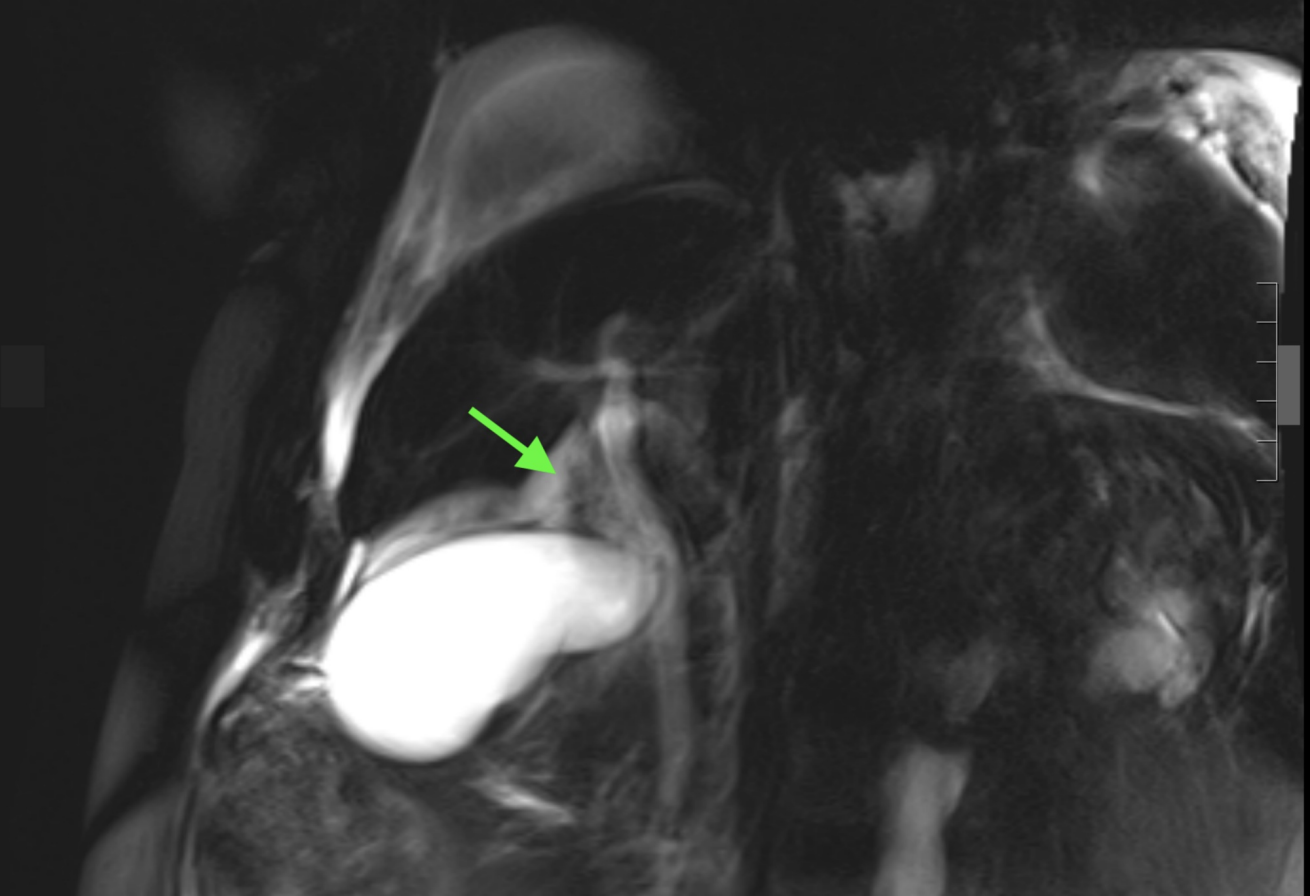
Magnetic resonance cholangiopancreatography (MRCP) of the abdomen MRCP showing moderate pericholecystic and perihepatic fluid collection (arrow).

MRCP revealed moderate pericholecystic and perihepatic fluid collection (Figure 3).

**Figure 4:**
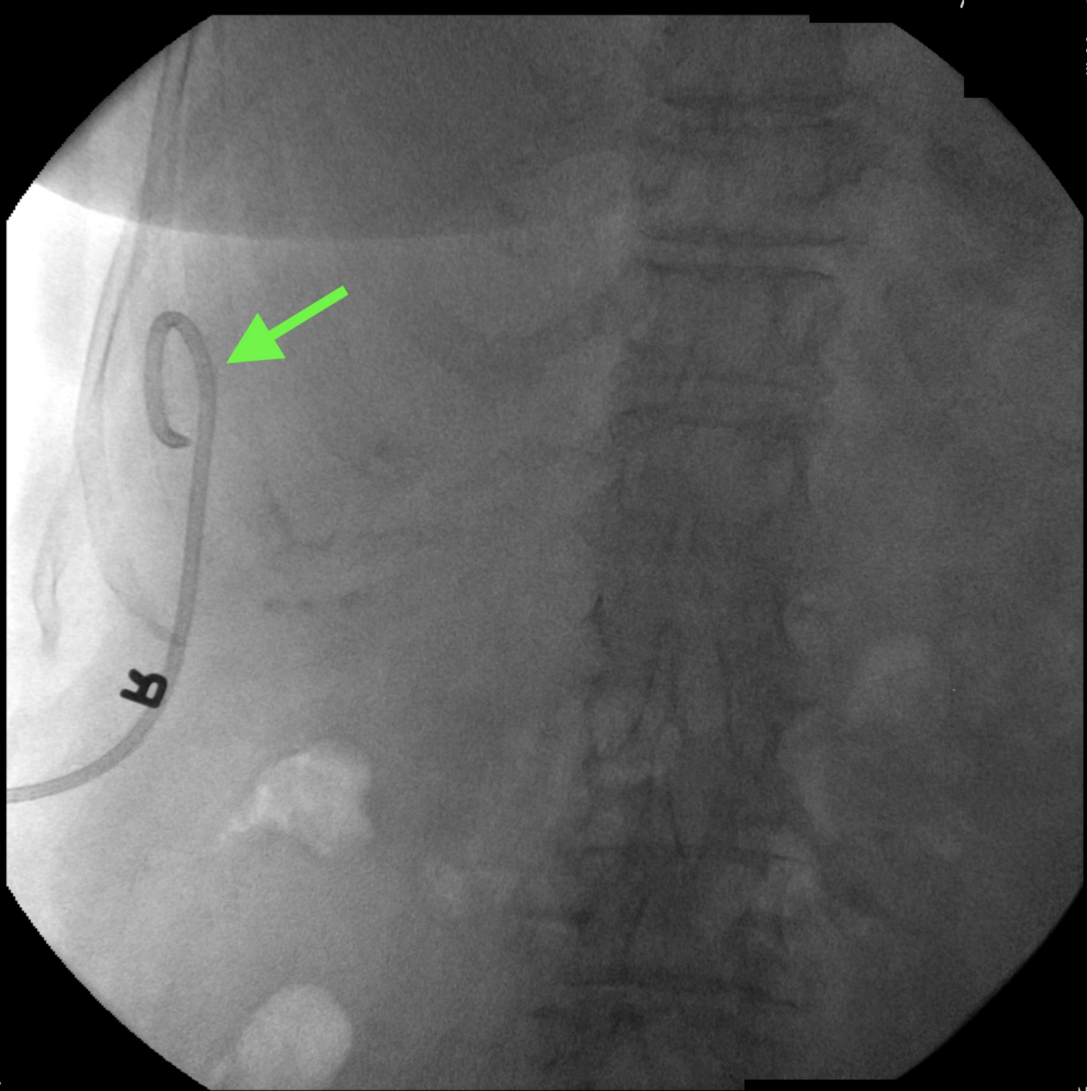
Cholangiogram Cholangiogram showing a catheter in place (arrow) after percutaneous drainage of biloma.

A cholangiogram demonstrated a perihepatic biloma, which was drained under the guidance of fluoroscopic imaging (Figure 4).

**Figure 5:**
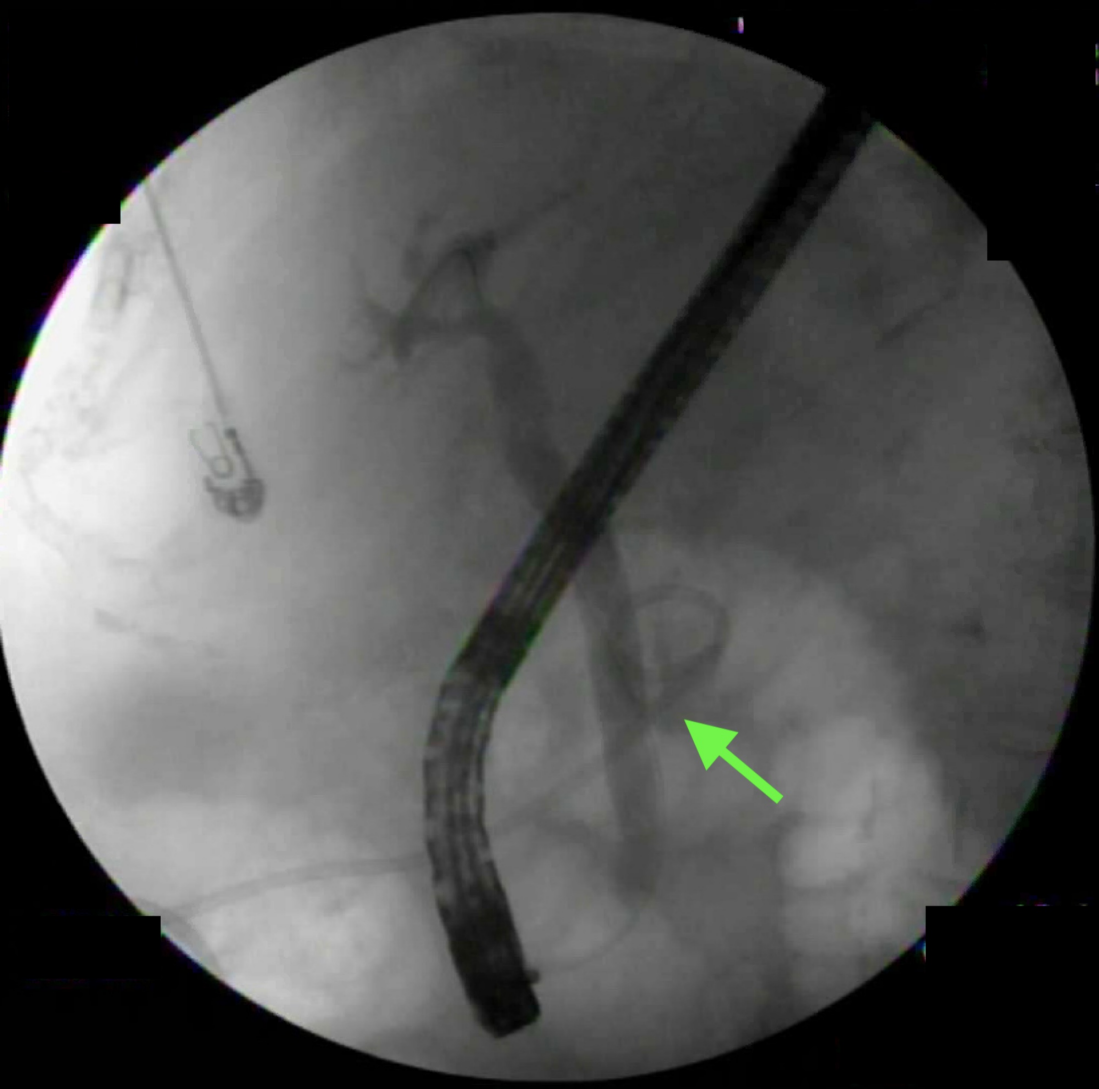
Endoscopic retrograde cholangiopancreatography (ERCP) ERCP showing dilated bile duct with peribiliary duct collection at the site of percutaneous biliary drainage catheter (arrow).

On endoscopic retrograde cholangiopancreatography (ERCP), there was no clear evidence of contrast extravasation; however, a blush of contrast at the junction of the cystic duct and the common hepatic duct was seen, which correlated with the location of biloma noted on the HIDA scan (Figure 5).

**Figure 6:**
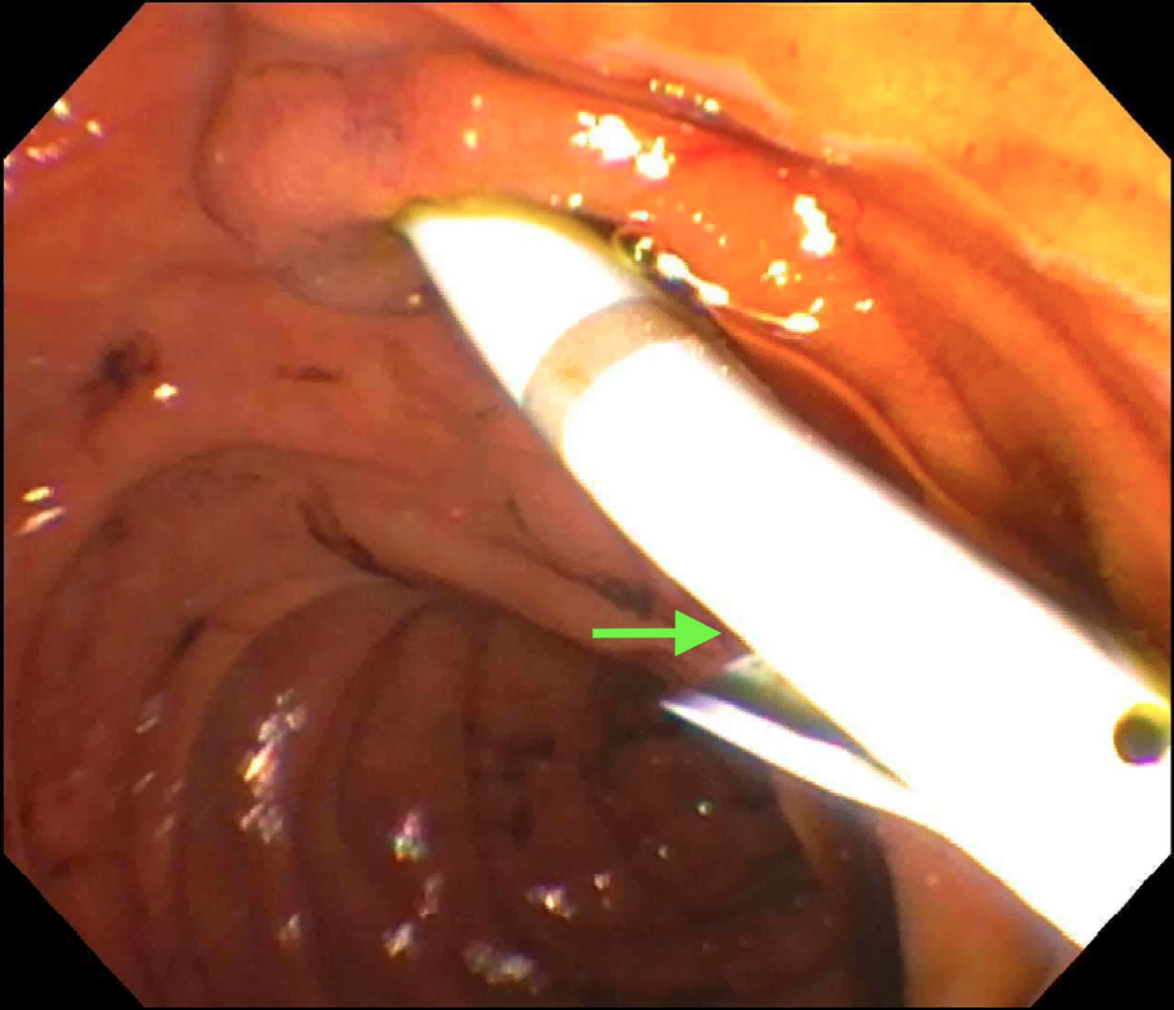
Endoscopic retrograde cholangiopancreatography (ERCP) ERCP-guided plastic stent (arrow) placed in the common bile duct for drainage of bile.

Since there was clear evidence of bile in the percutaneous drain with no clinical or endoscopic evidence of the bile leak, a 10-French plastic stent was placed in the CBD across the junction of the bile duct with the cystic duct where a blush of contrast was seen on ERCP (Figure 6).

Thereafter, the patient’s symptoms completely resolved, and she was discharged home. Upon follow-up five weeks after the intervention, a repeat ERCP showed that the bile leak had resolved, and the plastic stent was removed.

## Discussion

A biloma was first reported in 1979 by Gould and Patel as an encapsulated extrahepatic biliary collection [8]. The term biloma was extended to include an encapsulated bile collection outside the biliary tree, either extrahepatic or intrahepatic [9]. SBL is an etiology of biloma that may result in delayed diagnosis and intervention due to the rarity with which it is encountered in clinical medicine. The pathophysiology of SBL remains to be elucidated; however, persistently increased pressure in the intra- or extrahepatic biliary duct, biliary obstruction, or infarction are the suggested risk factors of SBL, resulting in biloma formation [10,11]. The clinical presentation of biloma is variable from non-specific abdominal pain to severe biliary sepsis, with the severity depending upon the location, size, and underlying etiology of the biloma [12,13]. Bilomas with traumatic or postsurgical etiologies are usually larger in size and these patients usually present earlier to seek medical attention due to more readily seen symptoms [9]. The patient described in this case report presented with abdominal pain and a few episodes of non-biliary, non-bloody vomiting, with elevated lipase indicating acute pancreatitis in the absence of radiological findings of pancreatitis. However, in this case, making the diagnosis of biloma was challenging due to the absence of typical risk factors, such as history of abdominal trauma, hepatobiliary interventions, or abdominal surgeries. Hence, one should be diligent to include SBL in the differential diagnosis for epigastric or RUQ abdominal pain, even without the typical risk factors being present or elicited on history. 

The diagnosis is based on the clinical presentation and more so, radiologic abdominal imaging. The US of the abdomen is the ideal initial imaging modality in the evaluation of RUQ pain, due to its non-invasiveness and rapid assessment. However, smaller bilomas can be missed prompting the need for further imaging, such as abdominal CT scan or MRCP, if symptoms persist despite a negative US. HIDA scan is the most effective diagnostic imaging modality in the identification of bile leak. In our case, HIDA scan confirmed the presence of bile leakage into the peritoneum when US did not and demonstrated a perihepatic biloma in the absence of typical history and clinical features. This prompted the next step of a cholangiogram and percutaneous drainage of biloma. The imaging-guided percutaneous aspiration of the biliary collection has both diagnostic and therapeutic roles in the management of biloma. It provides the fluid for biochemical analysis, as well as helping in the differentiation of a biloma from bilhemia, angioma, abscess, cystic lesions, lymphocele, seroma, or hematoma. The complete resolution of smaller bilomas may result with imaging-guided percutaneous drainage. A spontaneous resolution of bilomas with diameter 4 cm or higher is rare [3]. Untreated symptomatic patients and those with large bilomas (4 cm or greater diameter) are at risk for severe complications, such as biliary peritonitis, sepsis, pancreatitis, necrotizing fasciitis, abdominal abscess, gastrointestinal bleeding, pulmonary embolism, and respiratory failure [3].

Asymptomatic patients with smaller sized bilomas can be managed conservatively [12,14]. Symptomatic patients and those with a larger sized biloma are usually managed with percutaneous drainage, which eliminates the need for surgical drainage and the complications [12,15]. Follow-up US may be considered for patients undergoing drainage or surgery, to evaluate biloma resolution, and in those who are conservatively treated in order to document lesion stability [15]. EUS and ERCP are increasingly utilized in the diagnosis and management of spontaneous biloma with higher safety and technical feasibility than percutaneous drainage [16]. ERCP is a minimally invasive diagnostic tool, as well as a therapeutic modality, which is indicated in spontaneous active bile leak, recurrent extrahepatic biloma after percutaneous drainage, and in poor surgical candidates [9,13,17]. ERCP-guided placement of a biliary stent across the site of leak may result in complete resolution [13]. We used a combined approach with percutaneous drainage and ERCP-guided biliary stenting, which resulted in the complete resolution of symptoms in our patient. Emergent surgical exploration is required in hemodynamically unstable patients as septic shock, biliary peritonitis, biliopleural fistula, or bilhemia can increase the risk of mortality more rapidly. 

## Conclusions

Biloma should be included in the differential diagnosis of RUQ abdominal pain given the paucity of diagnosis-specific symptoms in some patients. A combined approach of fluoroscopic-guided biloma drainage and ERCP-guided biliary stent placement across the site of the biliary leak may have a higher success rate in the complete resolution of a spontaneous biliary leak and recurrent biloma formation than either approach alone. This should be preceded by the suitable imaging technique to appropriately identify the source of leak early on, thereby allowing the potential for less invasive approaches to promote resolution and overall improved outcomes. 
